# Tracking Australian Hajj Pilgrims’ Health Behavior before, during and after Hajj, and the Effective Use of Preventive Measures in Reducing Hajj-Related Illness: A Cohort Study

**DOI:** 10.3390/pharmacy8020078

**Published:** 2020-05-04

**Authors:** Amani Salem Alqahtani, Mohamed Tashani, Anita Elizabeth Heywood, Abdulrahman Bader S. Almohammed, Robert Booy, Kerrie Elizabeth Wiley, Harunor Rashid

**Affiliations:** 1 National Centre for Immunisation Research and Surveillance (NCIRS), The Children’s Hospital at Westmead, Westmead, NSW 2145, Australia; as.qahtani@sfda.gov.sa (A.S.A.); mohamed.tashani@health.nsw.gov.au (M.T.); robert.booy@health.nsw.gov.au (R.B.); 2 Sydney School of Public Health, Faculty of Medicine and Health, The University of Sydney, Sydney, NSW 2006, Australia; kerrie.wiley@sydney.edu.au; 3 Saudi Food and Drug Authority, Executive Department of Research and Studies, Riyadh 22332, Saudi Arabia; 4 Discipline of Child and Adolescent Health Children’s Hospital at Westmead Clinical School, the Faculty of Medicine and Health, The University of Sydney, Sydney, NSW 2145, Australia; 5 Department of Paediatrics, Faculty of Medicine, University of Tripoli, Ain Zara 13275, Libya; 6 School of Public Health and Community Medicine, The University of New South Wales, Sydney, NSW 2052, Australia; a.heywood@unsw.edu.au; 7 College of Medicine, Imam Muhammad Ibn Saud Islamic University (IMSIU), Riyadh 13317, Saudi Arabia; abalmohammed@imamu.edu.sa; 8 Marie Bashir Institute for Infectious Diseases and Biosecurity, School of Biological Sciences and Sydney Medical School, University of Sydney, Sydney, NSW 2145, Australia; 9 WHO Collaborating Centre for Mass Gatherings and High Consequence/High Visibility Events, Flinders University, Adelaide, SA 5000, Australia

**Keywords:** facemask, Hajj, hand hygiene, health behavior, infectious diseases, mass gathering

## Abstract

This study assessed Australian Hajj pilgrims’ knowledge, attitude and practices throughout their Hajj journey to understand their health behaviors, use of preventative measures and development of illness symptoms. A prospective cohort study with data collection at three phases (before, during and after Hajj) was conducted among Australian pilgrims between August and December 2015. Baseline data were collected from 421 pilgrims before Hajj, with 391 providing follow-up data during Hajj and 300 after their home return. Most participants (78% [329/421]) received one or more recommended vaccines; travel agents’ advice was the main factor affecting vaccination uptake. Most participants (69% [270/391]) practiced hand hygiene with soap and sanitizers frequently, followed by disposable handkerchief use (36% [139/391]) and washing hands with water only (28% [111/391]). During Hajj 74% (288/391) of participants reported one or more illness symptoms, 86% (248/288) of these symptoms were respiratory. Cough was less often reported among pilgrims who received vaccinations, cleaned their hands with soap or alcoholic hand rubs, while a runny nose was less common among those who frequently washed their hands with plain water but was more common among those who used facemasks. This study reveals that most Australian Hajj pilgrims complied with key preventative measures, and that tour group operators’ advice played an important role in compliance. Pilgrims who were vaccinated and practiced hand hygiene were less likely to report infection symptoms.

## 1. Introduction

The Hajj pilgrimage attracts over two million pilgrims every year to Saudi Arabia, making it one of the biggest annual human mass gatherings on the planet [1]. Consequently, with such large numbers of pilgrims from around the world in close proximity to one another, there is amplified risk of transmission of infectious diseases among those present [2]. To minimize the risks, Saudi Ministry of Health (MoH) require a valid vaccination certificate against meningococcal disease for all pilgrims, plus yellow fever and polio vaccines for pilgrims coming from or transiting through endemic countries. Additionally, vaccinations against influenza, diphtheria, pertussis, tetanus, mumps and measles are recommended, particularly for pilgrims susceptible to more severe disease [3]. Other relatively inexpensive preventative measures such as hand hygiene and facemask use are also recommended [4]. Studies have consistently shown that there is an extensive variation in vaccine uptake among pilgrims based on their country of origin, demographics and vaccines received. Similarly, hand hygiene and other preventative practices also vary among pilgrims, making it more difficult for researchers to ascertain whether vaccine uptake and health behaviors overall have improved in comparison to previous years or studies [5,6,7]. Although there is some research linking knowledge, attitudes, and beliefs of Hajj pilgrims to their use of preventive measures, no studies have explored their knowledge, preparedness and preventive practices over the course of their travel (i.e., following the same cohort before, during and after Hajj) [5,8,9,10,11]. To address these research gaps, we conducted a cohort study to explore Australian Hajj pilgrims’ knowledge about the risk of diseases during Hajj, assess their preparedness and use of preventive measures at three times points (before, during and after Hajj), investigate the factors affecting their preventive health behavior, and determine the number of reported infections during and after Hajj.

## 2. Materials and Methods

### 2.1. Study Population

This was a prospective cohort study undertaken between August and December 2015 among Australian Hajj travelers aged 18 years or older. The participants were residents of Greater Sydney, New South Wales, Australia. This region was chosen because it contains the largest Muslim population in New South Wales, Australia. The participants were assessed before, during and after returning from their pilgrimage to Makkah, Saudi Arabia. This study was approved by the University of Sydney Human Research Ethics Committee (HREC) (Project No: 2014/599).

### 2.2. Recruitment Methods

All overseas pilgrims travelling to Saudi Arabia require a visa which can only be acquired through approved Hajj travel agents [12]. These tour operators play a pivotal role in pilgrims’ preparation leading up to Hajj, relaying important travel instructions prior to departure through ‘pre-Hajj seminars’ which almost all prospective pilgrims attend [13]. This made these seminars an ideal recruitment points for a representative sample. The list of accredited Australian Hajj tour operators and their addresses were collected from the Saudi Embassy in Canberra, Australia. A selection of participating travel agencies was decided on the basis of the number of Hajj visas allocated for a given tour operator and the operators with the highest visa quotas were approached first.

Three questionnaires were used for data collection: (1) a pre-Hajj questionnaire completed prior to departure; (2) a ‘Hajj diary’ completed daily over six days during the peak Hajj period; and (3) a post-Hajj questionnaire, completed upon return to Australia. The surveys were mainly in English, with the availability of Arabic, Turkish and Urdu translations for those who preferred it.

### 2.3. Data Collection

#### 2.3.1. Pre-Hajj Survey

We attended eleven seminars held by the Hajj travel agents in Sydney from 1 August to 6 September 2015 to explain our research. Once pilgrims consented to participate in the study, their characteristics were obtained using a self-administered questionnaire documenting socio-demographic details, their travel itineraries, vaccination records, and presence of chronic medical conditions such as hypertension, diabetes, bronchial asthma and hyperlipidemia. Barriers to, and facilitators of vaccine uptake and data on risk perception of diseases at Hajj, such as influenza, pneumonia, blood borne infections and their preparedness for using preventive measures during Hajj were also collected. We did not ask about the receipt of meningococcal vaccine because our previous survey showed that all Australian pilgrims receive this as a mandatory visa requirement [13].

#### 2.3.2. ‘During Hajj’ Survey

We travelled to Makkah, Saudi Arabia, during Hajj period (15–30 September 2015) and identified and met the study participants upon their arrival in Mina, Greater Makkah. The participants were given a diary for self-completion daily over the six consecutive days of Hajj (the peak Hajj days). They were followed from 21–26 September 2015. Each participant was asked to record the following in the diary for each day: actual use of preventative measures including, facemask use, hand sanitizer use, hand washing after touching an ill person and use of disposable handkerchiefs. As the data were collected electronically, no respondent could submit their responses with vitally important information missing. Any respondent who used a preventive measure ≥5 days during the peak Hajj days considered to be ‘frequently compliant’, those who used the preventive measure <5 days were considered to be ‘infrequently compliant’ and those who did not use the preventive measure at all were considered ‘non-compliant’. Any development of symptoms suggestive of respiratory infection including cough, subjective fever sore throat, rhinitis and other symptoms including vomiting, diarrhea and nausea was also recorded. We considered those who complained of cough, subjective fever and sore throat to meet the criteria of influenza-like illness (ILI).

#### 2.3.3. Post-Hajj Survey

The respondents participated in a computer-aided telephone interview (CATI) seven to 10 days after their coming back to Australia (until 26 December 2015). They were asked about any development of symptoms suggestive of respiratory infection such as cough, sore throat, runny nose, fever, and also about some constitutional or gastrointestinal symptoms like vomiting, diarrhea and nausea after their home return from Hajj. Facilitators of and barriers to using preventive measures during Hajj were also explored. For each participant we made three call attempts before being classified as lost to follow-up.

### 2.4. Sample Size

A consecutive sampling strategy was used to ensure a representative sample of Hajj pilgrims living in NSW. Based on results from previous research, and considering an error margin of 5% to be acceptable for this survey, a sample size of 350 pilgrims was deemed to be sufficient. Considering a loss to follow-up rate of 20%, 420 participants were targeted, representing 12% of Australian pilgrims attending Hajj in 2015.

### 2.5. Data Analysis

Statistical analysis was performed using the SPSS v.23.0 (SPSS, Inc., Chicago, IL, USA). Chi-squared tests and Pearson correlation were used to assess variables and establish associations and correlations. Factors with *p* values < 0.25 in univariate analysis were entered into multivariable regression models. Binary logistic regression, using the backward Wald method, controlling for factors was used to investigate variables related to health behavior. *p* values less than 0.05 were considered statistically significant in multivariable models. A three-point Likert scale was used to measure the pilgrims’ perception about the risk of diseases during Hajj and the effectiveness of the preventive measures.

## 3. Results

### 3.1. Demographics

**Figure 1:**
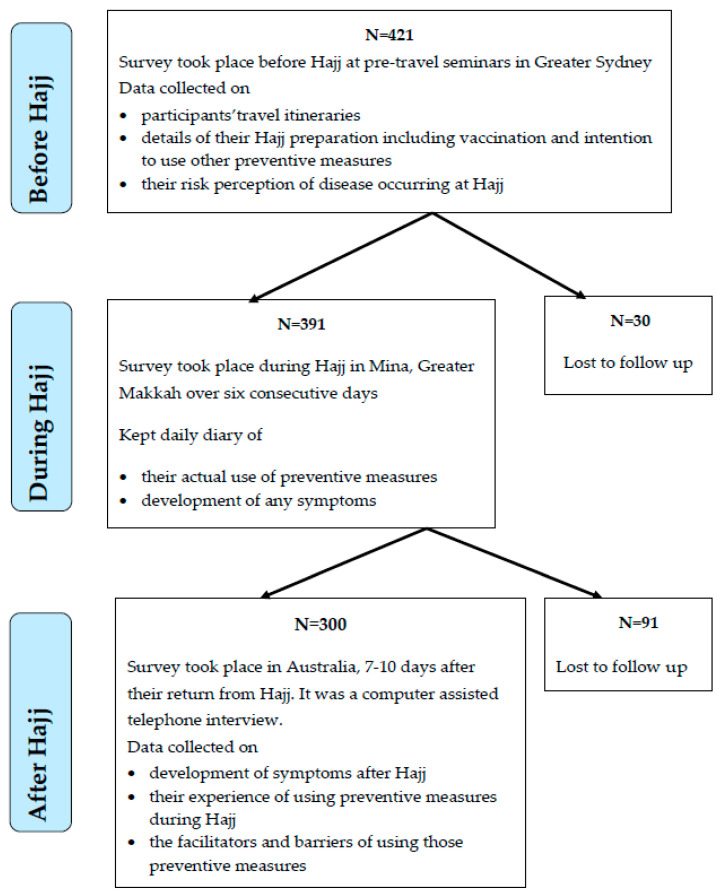
Flow chart of recruitment, participation and dropout rates.

A total of 421 pilgrims were recruited in the first stage of the study (before Hajj); out of those, 391 (93%) were followed during Hajj; and finally, 300 (71%) were reached after their return to Australia (Figure 1). Of 421, 46% were female and their mean age was 42.2 (standard deviation [SD] ± 11.2) years.

Over a quarter (28%) reported having one or more pre-existing medical conditions. Over one third of participants (39%) had a university degree or higher qualification and two thirds (66%) were employed. The pilgrims intended to stay in Saudi Arabia for a median of 25 (range: 10–45) days, and the majority (81%) were attending Hajj for the first time. The participants’ further demographic details are presented in Table 1.

### 3.2. Vaccine Uptake

The majority of participants (78%, [329/421]) received one or more recommended vaccines (i.e., vaccines that are recommended but not compulsory); of those, 43% (180/421) received only the influenza vaccine, 33% (139/421) received influenza plus other recommended vaccines while the remaining 2% (10/421) received only “other than influenza” vaccines. Overall, the coverage of influenza, pneumococcal and pertussis vaccine was, respectively, 76% (319/421), 25% (107/421) and 21% (88/421) (Table 2). On the other hand, 22% (92) did not receive any recommended vaccine.

### 3.3. Influenza and Pneumococcal Vaccination Rate Among ‘at Risk’ Individuals

Thirty-one percent (129/421) were in an at-risk category; of those, 20% (26/129) were aged ≥65 years and 80% (103/129) aged <65 but had one or more chronic medical condition (e.g., diabetes). The influenza vaccination rate among at-risk group was 76% (98/129), while the uptake of pneumococcal vaccine was 21% (27/129). No significant differences of influenza and pneumococcal vaccine uptake were noted between ‘at risk’ and not ‘at risk’ groups.

### 3.4. Barriers, Facilitators and Associated Factors of Vaccine Uptake

Participants reported different sources of vaccination advice including, Hajj tour operators (57%, [189/329]), general practitioners (GPs) (27% [90/329]), family members and friends with previous Hajj experience (13% [44/329]), and special websites (e.g., Smarttraveller and Saudi MoH) (2% [6/329]). Fear of falling ill during Hajj was the most cited reason (65% [212/329]) for receiving the vaccines (Table 2). Conversely, being unaware of the recommended vaccines (49% [45/92]) was the main reason for non-receipt of the vaccines (Table 2). Multivariate analysis showed that being aged between 36 and 64 years was significantly associated with the receipt of recommended vaccines compared to being aged ≤35 years or ≥65 years (adjusted odds ratio [aOR] = 2.1, 95% confidence interval [CI] = 1.2–3.6, *p* < 0.01). Moreover, those who had a university qualification or higher education were more likely to receive vaccine than those who had lower level of education (aOR = 4.1, 95% CI = 1.2–13.1, *p* < 0.01).

### 3.5. Risk Perception about Diseases

Before departing for Hajj, pilgrims reported being concerned about influenza (66% [278/421]), diarrhea (65% [275/421]), food poisoning (63% [265/421]), pneumonia (56% [234/421]), skin ailments (55% [228/421]) and blood borne diseases (52% [219/421]). Those who were very concerned about blood borne diseases (aOR = 2.1, 95% CI = 1.1–4.3, *p* = 0.02) and pneumonia (aOR = 1.8, 95% CI = 1.1–3.2, *p* < 0.01) were twice as likely to receive hepatitis B and pneumococcal vaccines respectively. Yet, no association was found between the level of concern about influenza and the receipt of influenza vaccine.

### 3.6. Non-Pharmacological Preventive Measures

#### 3.6.1. Intention to Use Non-Pharmacological Measures (before Hajj)

Before Hajj, participants reported their intention to use non-pharmacological preventive measures while at Hajj; the majority (73%, [306/421]) intended to use hand washing with soaps followed by the use of disposable handkerchiefs (72% [302/421]). Conversely, a lower proportion said they planned to use hand hygiene with alcoholic hand rubs (40% [167/421]) and use a facemask (38% [161/421]). On multivariate analysis, those aged <65 years (aOR = 2.2, 95% CI = 1.3–3.8, *p* < 0.01) and those who were worried about developing pneumonia at Hajj (aOR = 2.07, 95% CI = 1.3–3.1, *p* < 0.01) were more likely to accept using a facemask during Hajj, compared with those who were not. Moreover, those who were worried about diarrhea during Hajj were more likely to plan to wash their hands with soap and sanitizers at Hajj (aOR = 2.3, 95% CI = 1.2–4.1, *p* < 0.01). Those performing Hajj for the first time were more likely to plan to use alcoholic hand rub (aOR = 2.1, 95% CI= 1.2–3.6, *p* < 0.01) and disposable handkerchiefs at Hajj (aOR = 1.9, 95% CI = 1.01–3.6, *p* = 0.04).

#### 3.6.2. Actual Use of Non-Pharmacological Measures during Hajj

During Hajj, the majority of participants (69%, [270/391]) practiced hand hygiene with soap and sanitizers frequently; 36% (139/391) used a disposable handkerchief, and 28% (111/391) washed their hands with water only (Table 3). In addition, 19% (76/391) of pilgrims used antibiotics during Hajj. There was no significant association between pilgrims’ intended use of non-pharmacological measures and their actual use during Hajj (Table 4).

In multivariate analysis, no demographic factors were associated with using hand hygiene with soap and sanitizers, but those who were concerned about developing pneumonia during Hajj (aOR = 2.1, 95% CI = 1.1–4.3, *p* = 0.04) were more likely to practice hand hygiene with soap and sanitizers during Hajj compared to those who were not concerned. Those aged ≥65 years (aOR = 3.1, 95% CI = 1.1–8.8, *p* = 0.02) were more likely to practice hand hygiene with alcoholic hand rubs than those aged <65 years. Moreover, males were more likely to wash their hands with water only (aOR = 1.9, 95% CI = 1.2–2.9, *p* < 0.01) and wash hands after touching an ill person (aOR = 2.4, 95% CI = 1.3–4.6, *p* < 0.01) but were less likely to use disposable handkerchiefs (OR = 0.4, 95% CI = 0.3–0.7, *p* < 0.01) compared to females.

#### 3.6.3. Barriers and Facilitators to the Use of Facemasks and Hand Hygiene during Hajj

After Hajj, among those who used facemasks during Hajj (irrespective of the frequency of use) the major reasons that influenced their decision to use facemasks included protection from disease (42%, [54/127]), protection from air pollution (35%, [44/127]) and to protect others from diseases such as family, friends or roommates (23%, [29/127]). Conversely, reasons for non-compliance included breathing discomfort (48%, [128/264]), belief that it was not necessary (35%, [92/264]) and a feeling of isolation from others (17%, [44/264]). Of those who practiced hand hygiene with soap and sanitizers (irrespective of frequency of use), reasons for use included a belief in its effectiveness in preventing infectious diseases (40%, [144/362]), habitual practice (37%, [134/362]) and ease of implementation (23%, [83/362]).

### 3.7. Incidence of Hajj-Related Illnesses

#### 3.7.1. During Hajj

**Figure 2:**
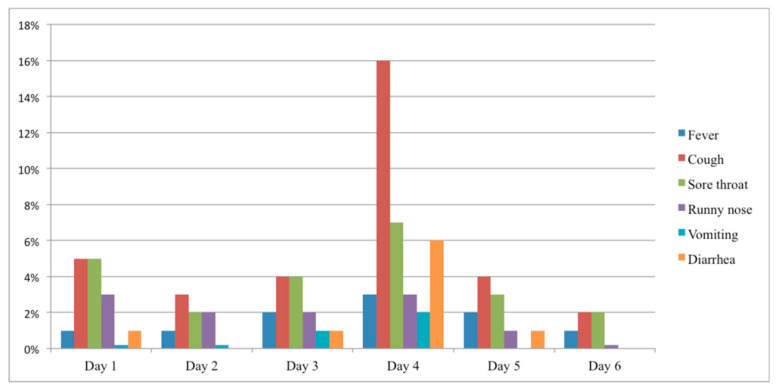
The reported onset of symptoms by days of Hajj among the Australian adult Hajj pilgrims.

On the first day of Hajj, some pilgrims were symptomatic; 5% had a cough, 5% had a sore throat, and 3% had a runny nose. However, on the 4th day up to 16% became symptomatic. The reported onset of daily symptoms among pilgrims during the peak days of Hajj is presented in Figure 2.

Overall, 74% (288/391) of participants reported one or more illness symptoms throughout the Hajj journey. Eighty-six percent (248/288) of these symptoms were respiratory, including cough, 45% (176/391); sore throat, 44% (171/391); runny nose, 26% (103/391); and fever, 15% (59/391). ILI was only reported among 10% (40/391) of participants. Additionally, 16% (64/391) reported diarrhea, 12% (46/391) nausea and another 5% (21/391) reported vomiting. Twenty six per cent (103/391) of respondents did not report any symptom during Hajj.

#### 3.7.2. After Hajj

Over half (52% [157/300]) reported symptoms after returning from Hajj; of those, 37% (111/300) reported cough (41%, [45/111] of these also had cough during Hajj), 25% (76/300) sore throat (43%, [33/76] of them reported sore throat during Hajj), 16%, (47/300) runny nose (30%, [14/47] reported runny nose during Hajj) and 11% (32/300) had fever (16%,[5/32] had fever during Hajj). ILI was reported in 10% (29/300) of participants; of those, 10% (3/29) also reported ILI during Hajj. Moreover, 5% (15/300) reported diarrhea, 3% (8/300) nausea and another 3% (8/300) reported vomiting.

#### 3.7.3. Effectiveness of Preventive Measures in Reducing Hajj-Related Illnesses

As shown in Table 5, cough was less likely to occur among those who were vaccinated against influenza and those who used alcoholic hand rubs. Runny nose was less likely to occur who frequently washed their hands with plain water but was more common among those who used facemasks. Vomiting was less likely to be reported among those who washed their hands frequently with soap and sanitizers during Hajj, but more common in those who used disposable handkerchiefs. No association was found between use of any of the preventive measures and reduction in fever and sore throat.

## 4. Discussion

This cohort study captured and compared the health behavior, knowledge, attitudes and practices of Australian Hajj pilgrims regarding preventative measures against communicable diseases throughout the course of Hajj travel (before, during and after the journey). Vaccinated pilgrims and those who washed their hands and used alcohol rubs were less likely to develop respiratory symptoms during Hajj.

Influenza vaccination coverage was relatively higher (76%) among Australian pilgrims, compared to that reported in studies from other countries, but was lower compared to that in Australian pilgrims in 2014 (80%) and in 2013 (83%) [5,14]. In contrast, coverage of other recommended vaccines, such as the pneumococcal vaccine, was suboptimal (25%), as has been reported in previous studies involving Australian Hajj pilgrims and other international pilgrims [15,16]. The coverage of influenza vaccine among pilgrims with high-risk conditions was 76%, while that of pneumococcal vaccine was only 21%; compared to the influenza vaccination coverage among pilgrims with high-risk conditions from other countries, Australian Hajj pilgrims had a higher vaccination rate [5]. The low pneumococcal vaccine uptake is a concern because pneumococcal diseases, including invasive pneumococcal disease, are major causes of morbidity and mortality in the extremes of age and in individuals with chronic medical conditions worldwide, including Hajj pilgrims [17,18]. Of note, while the influenza vaccine is highly recommended by the Saudi MoH for Hajj attendance, particularly for pilgrims aged ≥65 years [4], there is no formal recommendation regarding the pneumococcal vaccine.

This study found that a large number of participants were willing to use non-pharmacological preventative measures to reduce the prevalence of illnesses during Hajj. However, there was no significant correlation between their intention to use measures and their actual use during Hajj. Although the level of concern for pneumonia and diarrhea was higher among first time Hajj goers and pilgrims <65 years of age, these factors were not shown to be significant in their actual use of preventive measures during Hajj. Pilgrims who were concerned about catching diseases and those who joined Hajj for the first time were more likely to accept the use of non-pharmacological measures before Hajj. However, these intentions were not associated with actual use of preventive measures. Previous studies have not explored if demographic factors were associated with an intention to use non-pharmacological protective measures before travel and their actual use during Hajj. Nonetheless, earlier studies did conclude that health education prior to departure was significantly associated with greater compliance with preventative practices, particularly the use of facemasks and hand sanitizers [19,20,21,22].

**Figure 3:**
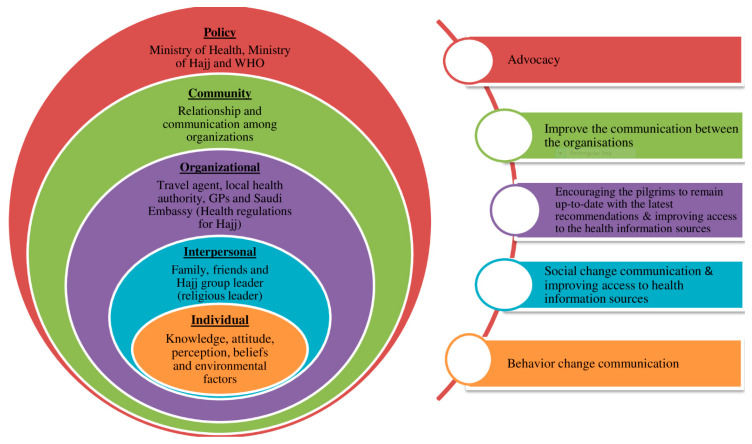
Applied Social-Ecological model on the factors affecting the health preventive measures uptake among Hajj pilgrims and the corresponding promotion approach.

Demographic factors had minimal association with pilgrims’ health behavior such as vaccine uptake, and the factors associated with pilgrims’ willingness to use preventive measures were not associated with their actual use during Hajj. Consequently, these findings pose questions of other possible causes that might affect pilgrims’ behaviors during Hajj. In this study, several factors were identified as influencing pilgrims’ health behavior, including disease risk perception, awareness of recommendations, the influence of people around them, age and medical history, the source of their travel health advice, and the lived experience of using preventive measures during Hajj. These factors can be stratified into five categories: individual, interpersonal, organizational, community-associated and policy-related. Relying on a single factor does not fully explain the interplay and dynamics between pilgrims’ health behaviors and their influencers; rather, consideration of multiple factors at different levels may help to understand these cross-cutting relationships. These dimensions fit within the ‘social-ecological model’ which emphasizes the connectivity and relationship among multiple factors affecting health behavior, as shown in Figure 3. The overlapping circles in the model illustrate how factors at one level influence factors at another level. This framework is based on evidence that no single factor can explain why only some people comply with preventive measures while remain influenced by multiple inter-related factors. The core of the model is at the individual level, surrounded by the outer four bands representing the interpersonal, organizational, community and policy levels (Figure 3). It is important to understand and find the most common or influential reasons and the link between these levels to enhance pilgrims’ health behavior uptake in regards to each measure. In addition, while some interventions such as vaccines are known to be clinically effective, their acceptability to pilgrims is a vital part of their overall effectiveness as a public health intervention. Effective behavioral change is only made when changes interconnect between the individual, community, organizational and policy levels. This includes addressing underlying and related factors such as individual beliefs, culture, and miscommunication between organizations as well as government policies. This can help promote lasting changes in practices; on an individual level, cultural and religious beliefs; in attitudes and perception; in communication between the health care providers such as GPs and the community; providing the service suppliers such as the travel agent with up-to-date health recommendations and also improving the communication between the international and local health policies (Figure 3) [23]. Future studies could be grounded in ‘social ecological’ theory, in order to further articulate the inter-level relationships between health behavior factors identified in this study, encouraging new insights to promote the health interventions among Hajj pilgrims.

During Hajj, about 63% of participants reported developing one or more respiratory symptoms; a cough and sore throat were the commonest symptoms similar to what was found in earlier studies involving Australian pilgrims who attended Hajj in 2014 [24], and among French, Iranian and Malaysian pilgrims who attended Hajj between 2003 and 2012 [25,26,27]. During Hajj, ILI was reported by 10% of pilgrims in this study, a similar rate was reported previously among Australian Hajj pilgrims in 2013 [28]. In other studies involving Hajj pilgrims the reported incidence of ILI varied (from 10% to 70%) [29]. The risk of disease was equally high among returning pilgrims, with 30% to 40% of them reporting cough, sore throat and runny nose, and 10% suffering from ILI as found in other studies [29,30,31]. Countries should continue to monitor the pilgrims and their contacts after the pilgrims’ return from Hajj to estimate the risk of post-Hajj infections.

Influenza vaccine effectiveness was studied in several studies. In two studies conducted in 2003 and 2012, a reduction in ILI was observed, while in a few other earlier studies no significant reduction was noted [5]. A synthesis of published and raw data from eleven Hajj years between 2005 and 2014 showed that the rate of ILI decreased among Hajj pilgrims as the vaccination rate increased (relative risk 0.2, *p* < 0.01) [32]. Subsequently, a ‘test-negative’ case-control study using data from individual Hajj years involving participants from multiple countries shows trivalent influenza has an effectiveness of 43.4% (95% CI 11.4% to 63.9%, *p* = 0.01) against laboratory-confirmed influenza [33]. The varying results may due to heterogeneity in defining ILI or may be because of ILI symptoms representing non-influenza infections or even due to confounding factors such as use of facemasks or hygienic interventions [30,31,34].

There are some limitations; 29% respondents failed to complete the study. This is a little higher than the expected loss to follow up (20%), however as this study was conducted over three months in three different settings (before, during and after Hajj) in two countries (Australia and Saudi Arabia), it was very challenging to follow all participants, some of whom may have taken a side trip to other countries at the end of the Hajj. A French study revealed that over a quarter of the pilgrims intended to delay their return to France after Hajj, a situation that makes difficult to ensure maximum follow up [35]. Information and recall bias may have occurred due to the anecdotal nature of some survey questions, especially those of the post-Hajj survey.

Although our results cannot be generalized to all pilgrims, this study is the first of its kind to assess pilgrims’ KAP continuously throughout the Hajj journey to understand their health behaviors, experience of using preventative measures and the development of acute respiratory infections, and other symptoms of infections. In the meantime, studies conducted in France and Malaysia have shown that compliance to some preventive measures such as hand hygiene and face mask use has increased, while the uptake of recommended vaccines including influenza and pneumococcal vaccines still remains low [36,37,38]; therefore, awareness campaigns should be continued to tackle respiratory infections including the ongoing COVID-19 pandemic that, thus far, has taken a toll of over 200,000 people across the world (as of 27 April 2020) and affected many Muslim countries including Saudi Arabia [39]. To mitigate the epidemic, the Saudi Arabian authority has already temporarily cancelled Umrah (minor pilgrimage) visit to Makkah [40]; the decision on whether this year’s Hajj pilgrimage (late July to early August) should be cancelled or not remains to be decided and may depend on the progress of the pandemic [41]. Our findings mean that preventive measures like hand washing and use of alcoholic hand rubs could be implemented readily during Hajj, and tour operators may play important roles in improving compliance.

## 5. Conclusions

In conclusion, this study reveals that most Australian Hajj pilgrims complied with key preventative measures, and that tour group operators’ advice played an important role in compliance. Pilgrims who complied with preventive measures were less likely to suffer from infection symptoms. Researchers and policy makers should work together to explore ways to educate the pilgrims and their tour operators about the importance of vaccination and using simple but inexpensive preventive measures, such as hand hygiene, that may halt the spread of highly contagious infectious diseases, e.g., COVID-19, pandemic influenza and drug-resistant pathogens in mass gatherings.

## Figures and Tables

**Table 1:** Demographic characteristics of recruited participants (*n* = 421).

Characteristic	*n* (%)
**Age groups**	
Mean	42.2 years
SD	± 11.2
**Gender**	
Male	229 (54)
Female	192 (46)
**Education**	
No formal education	23(5)
School certificate (year 10 equivalent)	75 (18)
High school certificate (year 12 equivalent)	98 (23)
Certificate/diploma	61(15)
University degree and higher degree	164 (39)
**Employments status**	
No	141(34)
Yes	280 (66)
*Self-employed*	79 (28)
*Full time*	140 (50)
*Casual*	25 (9)
*Part-time*	35 (13)
**Country of birth**	
Australia	128 (30)
Middle Eastern countries	141 (33)
Indian sub-continent	103 (24)
Southeast Asian countries	34 (8)
Others	15 (4)
Median years of stay in Australia *	21.5 years
**Chronic diseases**	
No	303 (72)
Yes	118 (28)
*Diabetes*	41(35)
*Asthma*	33 (28)
*High cholesterol*	30 (25)
*Hypertension*	28 (24)
*Heart diseases*	8 (7)
*Cancer*	3 (3)
*Other lung diseases*	2 (2)
*Chronic kidney disease*	2 (2)
*Immunosuppressive condition (e.g., HIV, long-term steroid use)*	1 (1)

* For those who were born overseas; SD standard deviation.

**Table 2:** Uptake of recommended vaccines and reported reasons for receipt and non-receipt among adult Australian Hajj pilgrims 2015 (*n* = 421).

Vaccine Name	*n* (%)
Seasonal influenza vaccine	319 (76)
Pneumococcal vaccine	107 (25)
Pertussis (whooping cough) vaccine	88 (21)
Hepatitis A vaccine	45 (11)
Hepatitis B vaccine	46 (11)
MMR (measles, mumps, and rubella) vaccine	33 (8)
dTP (diphtheria, tetanus, polio)	33 (8)
Typhoid vaccine	29 (7)
Polio vaccine	9 (2)
Vaccinated Reasons *	
I don’t want to get sick	212 (65)
The vaccine is effective in protecting me against diseases	64 (20)
If I get sick my Hajj worship could be jeopardised	63 (19)
By vaccinating, I can prevent passing diseases to others	45 (14)
I am at risk because I’m at risk/low immunity	20 (6)
Not Vaccinated Reason *	
I didn’t know about them	45 (49)
I don’t think I will get a disease at Hajj, I’m under “ALLAH’s” protection	21 (23)
I don’t need them because I’m not at risk	20 (22)
I rely on my own body’s immunity (healthy lifestyle)	10 (11)
The vaccines are too expensive, they should be free	4 (4)
I was afraid of having vaccine side effects	3 (3)

* Some pilgrims cited more than one reason.

**Table 3:** Australian Hajj pilgrims’ actual use of non-pharmacological measures during Hajj 2015 (*n* = 391).

Preventive Measures Use	Frequently *n* (%)	Infrequently *n* (%)	Non-Compliant *n* (%)
Facemask	76 (19)	51 (13)	264 (68)
Hand washing with antibacterial gel and soap	270 (70)	92 (23)	28 (7)
Use of alcoholic hand rub	61 (16)	23 (6)	306 (79)
Hand washing with plain water	111 (29)	81 (21)	198 (51)
Hand washing after touching an ill person	51 (13)	12 (3)	327 (84)
Use of disposable handkerchiefs	139 (36)	64 (16)	187 (48)

**Table 4:** Association between adult Australian Hajj pilgrims’ preparedness to comply with a preventive measure and their actual compliance during Hajj 2015 (*n* = 421).

	Intention *n* (%)	Actual Use “Yes” *n* (% *)	*p* Value
Facemask			
*No*	169 (40)	40 (24)	0.9
*Possibly*	91 (22)	22 (24)	
*Yes*	161 (38)	34 (21)	
Hand washing with antibacterial gel and soap			
*No*	55 (13)	41 (75)	0.1
*Possibly*	60 (14)	56 (93)	
*Yes*	306 (73)	243 (79)	
Hand washing with plain water			
*No*	92 (22)	45 (49)	0.06
*Possibly*	46 (11)	18 (39)	
*Yes*	283 (67)	103 (36)	
Use of alcoholic hand rub			
*No*	196 (47)	28 (14)	0.6
*Possibly*	58 (14)	9 (16)	
*Yes*	167 (40)	30 (18)	
Hand washing after touching an ill person			
*No*	87 (21)	11 (13)	0.5
*Possibly*	39 (9)	4 (10)	
*Yes*	295 (70)	44 (15)	
Use of disposable handkerchiefs			
*No*	85 (20)	25 (29)	0.2
*Possibly*	34 (8)	26 (76)	
*Yes*	302 (72)	120 (40)	

* Percentage of those who carried out their expressed intention regarding the intervention.

**Table 5:** Effectiveness of various preventive measures in reducing symptoms of Hajj-related illnesses.

Preventive Measures	Symptoms	Symptoms in Those Who Complied *n* (%) and Did not Comply with the Preventive Measure *n* (%)	aOR (95% CI) *p*
Hand washing with plain water	Fever	16 (9.4); 28 (12.8)	0.7 (0.4–1.4), 0.30
	Cough	56 (32.9); 92 (42)	0.7 (0. 5–1.0), 0.07
	Sore throat	68 (40); 103 (47)	0.8 (0.5–1.1), 0.17
	Runny nose	33 (19.4); 70 (32)	0.5 (0.3–0.1), 0.03
	Vomiting	6 (3.5); 8 (3.7)	1.0 (0.3–2.8), 0.07
Hand washing with soap and antibacterial products	Fever	39 (11.5); 5 (10)	1.2 (0.4–3.1), 0.76
	Cough	132 (38.8); 16 (32)	1.4 (0.7–2.5), 0.36
	Sore throat	145 (42.6); 26 (52)	0.7 (0.4–1.2), 0.22
	Runny nose	88 (25.9); 15 (30)	0.8 (0.4–1.6), 0.62
	Vomiting	13 (3.8); 1 (2)	2.0 (0.3–15.2), 0.64
Use of alcoholic hand rub	Fever	7 (10.3); 37 (11.5)	0.9 (0.4–2.1), 0.78
	Cough	18 (26.5); 130 (40.4)	0.4 (0.2–0.9), 0.02
	Sore throat	29 (42.6); 142 (44.1)	0.9 (0.6–1.6), 0.83
	Runny nose	17 (25); 86 (26.7)	0.9 (0.5–1.7), 0.77
	Vomiting	3 (4.4); 11 (3.4)	1.3 (0.4–4.8), 0.40
Hand washing after touching an ill person	Fever	7 (11.9); 37 (11.2)	1.1 (0.5–2.5), 0.89
	Cough	20 (33.9); 128 (38.7)	0.8 (0.5–1.5), 0.49
	Sore throat	24 (40.7); 147 (44.4)	0.9 (0.5–1.5), 0.59
	Runny nose	19 (32.2); 84 (25.4)	1.4 (0.8–2.5), 0.27
	Vomiting	3 (5.1); 11 (3.3)	1.6 (0.4–5.8), 0.51
Use of disposable handkerchiefs	Fever	24 (13.9); 20 (9.3)	1.6 (0.8–3.0), 0.16
	Cough	68 (39.3); 80 (37)	1.1 (0.7–1.7), 0.46
	Sore throat	80 (46.2); 91 (42.1)	1.18 (0.8–1.8), 0.42
	Runny nose	52 (30.1); 51 (23.6)	1.4 (0.9–2.2), 0.15
	Vomiting	10 (5.8); 4 (1.9)	3.3 (1.0–10.6), < 0.05
Facemask	Fever	13 (10.3); 31 (11.7)	1.3 (0.7–2.7), 0.43
	Cough	39 (31); 108 (40.9)	1.2 (0.7–1.9), 0.51
	Sore throat	39 (31); 131 (49.6)	0.9 (0.5–1.4), 0.48
	Runny nose	33 (26.2); 69 (26.1)	2.1 (1.2–3.6), < 0.01
	Vomiting	5 (4); 9 (3.4)	1.7 (0.6–5.3), 0.33
Influenza vaccine	Fever	34 (10.7); 1 (10)	1.2 (0.15–9.6), 0.88
	Cough	105 (32.9); 7 (70)	0.2 (0.1–0.9), 0.02
	Sore throat	133 (41.7); 5 (50)	0.8 (0.2–2.9), 0.77
	Runny nose	73 (22.9); 5 (50)	0.3 (0.1–1.2), 0.09
	Vomiting	10 (3.1); 1 (10)	0.3 (0.04–2.8), 0.30
Pneumococcal vaccine	Fever	10 (9.3); 25 (11.3)	0.8 (0.4–1.7), 0.55
	Cough	38 (35.5); 74 (33.3)	1.10 (0.7–1.8), 0.79
	Sore throat	39 (36.4); 99 (44.6)	0.7 (0.4–1.1), 0.11
	Runny nose	27 (25.2); 51 (23)	1.1 (0.6–1.9), 0.73
	Vomiting	5 (4.7); 6 (2.7)	1.7 (0.5–5.8), 0.38
